# Authors’ Response to Peer Reviews of “Raw, Unadulterated African Honey for Ulcer Healing in Leprosy: Protocol for the Honey Experiment on Leprosy Ulcer (HELP) Randomized Controlled Trial”

**DOI:** 10.2196/56442

**Published:** 2024-03-01

**Authors:** Sunday Udo, Pius Ogbu Sunday, Paul Alumbugu Tsaku, Israel Olaoluwa Oladejo, Anthony Meka, Linda Chinonso Ugwu, Motunrayo Ajisola, Joshua Akinyemi, Abiola Oladejo, Akinyinka Omigbodun, Sopna Mannan Choudhury, Jo Sartori, Onaedo Ilozumba, Sam Watson, Richard Lilford

**Affiliations:** 1 The Leprosy Mission Nigeria, Abuja, Nigeria; 2 German Leprosy and TB Relief Association/RedAid Nigeria, Enugu, Nigeria; 3 University of Ibadan, Ibadan, Nigeria; 4 University of Birmingham, Birmingham, UK

**Keywords:** leprosy, ulcers, wounds, honey, neuropathy, nerves, Africa, randomized controlled trial, RCT

*This is the authors’ response to peer-review reports for “Raw, Unadulterated African Honey for Ulcer Healing in Leprosy: Protocol for the Honey Experiment on Leprosy Ulcer (HELP) Randomized Controlled Trial.”*

## Round 1 Review

All the editorial comments are noted and carefully followed.

### Anonymous [1]

#### General Comments

*This is an excellent interventional protocol for a randomized controlled trial assessing honey as a potential ulcer therapeutic. Careful consideration has been made to avoid bias and ensure robust results. I would suggest a few things to consider (below) prior to publishing.*

#### Specific Comments

##### Major Comments

###### Background and Rationale

- *It is mentioned that 30% to 50% of people infected with leprosy have nerve damage. Be more specific here with “people”—is this a global estimate, American estimate, Nigerian estimate, etc?*

Response: We have now added that it is a global estimate according to a study by Napit et al [2].

- *The background may benefit from a more specific discussion of previous literature. If there is a significant systematic review on the topic—a quick summary of relevant findings in the background (or later in the discussion) would help to situate the rationale behind carrying out such a study.*

Response: We have worked on the literature review; however, we are also mindful of the word limits for this manuscript [3].

###### Study Setting

- *St. Benedict’s Tuberculosis and Rehab Hospital is owned by the Catholic Diocese. Do the authors suspect a potential religious bias in individuals who attend this hospital? Does this affect other social demographics and potentially skew generalizability?*

Response: We do not anticipate any bias associated with the having 1 of the 2 study sites be a Catholic Diocese–owned hospital. This is because the hospital is located in South-South Nigeria, where the citizens are predominantly Catholics. The St. Benedict’s hospital is also a well-recognized facility that offers services to people affected by leprosy in the southern region of Nigeria.

Similarly, the Chanchaga Hospital in Niger State, North Central Nigeria, sees most of it’s patients from the north. We hoped that having participants from the 2 sites will create some form of balance in our study.

- *I am not sure it is necessary to go into this much detail about the staffing compositions and facilities of each site. Consider truncating.*

Response: We have expunged the unnecessary details about staffing in the 2 facilities.

###### Additional Consent Provisions

- *The section mentions that the computer program will range-check information. Please specify which computer program.*

Response: The computer program (ie, Research Electronic Data Capture [REDCap]) is now specified in the manuscript.

###### Intervention Description

- *The honey is being obtained from local bee farmers in North Central Nigeria; however, it is unclear how the honey is being prepared prior to inclusion into the study. I understand it is being checked for botulism (which is great); however—I wonder—is the honey from difference farms being mixed together prior to use? Or is it possible that 1 dressing may be from 1 specific farm, etc? If so, is there a potential risk of interventional procurement bias? Meaning the honey from one farm may be better at wound healing then the honey from another farm? Just something to think about…*

Response: We have a single source for the honey used throughout the study. We try to maintain the integrity of the honey by not processing it other than to filter out the debris.

- *Be more clear about the function of the video recording. Will it also be used to test if assessors can distinguish between honey versus control?*

Response: The video recording at the start of the study is mainly for quality check, including the blinding of the assessors. Once the assessors are happy with the recorded procedure, the clinicians will be asked to proceed with the study.

###### Confidentiality

- *This section mentions that study forms containing personal identifier information will be kept secured and locked at trial site. Which trial site? Just 1? Or both? Please be more specific here.*

Response: The records are to be maintained at both trial sites. It has now been specified.

###### Other

- *Will you be collecting demographic data such as sex, gender, creed, socioeconomic status, level of schooling, etc? Would you consider stratifying results by any of these parameters?*

Response: Yes, all the demographic data are included in the baseline data collection on REDCap.

- *Additionally, will you be collecting information on leprosy status, that is, paucibacillary vs multibacillary leprosy or if the patient has progressed to the leprosy reaction stage (type 1 or type 2)? This information may be useful for downstream analysis and can be stratified for to avoid spectrum bias.*

Response: Yes, all the information and more are specified are in the data collection tool.

### Minor Comments

#### Background and Rationale

- *Edit sentence to read: “…and peripheral nerves, causing neuropathy and severe disability, consequently resulting in social exclusion and stigmatization.”*

Response: Done.

- *Edit sentence to read: “…new child cases [3], with a grade 2 disability rate of about 15% for the past…”*

Response: Done.

- *Edit sentence to read: “Thirty to fifty percent of…”*

Response: Done.

- *Edit sentence to read: “Ulcers usually occur in anesthetic feet, and will heal slowly with routine therapy, however have a tendency to recur [6].”*

Response: Done.

- *Edit sentence to read: “…documented report record in the Edwin Smith Papyrus…”*

Response: Done.

- *Edit sentence to read: ”…gathered and modified by the honeybee…”*

Response: Done.

- *Edit sentence to read: “…exudates, and possesses antimicrobial…”*

Response: Done.

- *Edit sentence to read: “…treatment of difference kinds of wounds, as researchers continue…”*

Response: Done.

- *Edit sentence to read: “…sizeable number of reports that show mixed levels…”*

Response: None.

- *Edit sentence to read: “…with only about 5% of patients reporting pain following dressing.”*

Response: Done.

- *The rest of the previous sentence “…and undocumented concern of botulism disease due to infection…” is unclear. Do the same 5% of patients also report concerns of botulism? Or is botulism a concern the authors have, and that has not been reported in previous literature? Either way I would make the botulism argument its own separate sentence that is more clear.*

Response: We have now separated the sentences to make it clearer for the readers to understand.

#### Study Setting

- *Is it “St. Benedict’s TBL” or “St. Benedit’s TBL”? Please correct all instances to 1 or the other. The first sentence under study setting uses “St Benedits.”*

Response: The correction is done.

- *In “…is a TB and leprosy…” please type out “Tuberculosis” on first use with “(TB)” in quotes as per other abbreviations.*

Response: Done.

#### Eligibility Criteria

- *Edit sentence to read: “…in the intervention group, all ulcers – not just the one…”*

Response: Done.

- *Edit sentence to read: “Routine swabs will be taken, but the interpretation of…”*

Response: The sentence has been modified in response to a comment by another reviewer.

#### Additional Consent Provisions

- *Edit sentence to read: “Photograph of the ulcers will be…”*

Response: Done.

#### Relevant Concomitant Care

- *Edit sentence to read: “…bearing and the level of activity of patients might…”*

Response: Done.

#### Recruitment

- *Edit sentence to read: “…identified by the on-site clinical…”*

Response: Done.

#### Plans for Assessment

- *Edit sentence to read: “…database managers at the University of…”*

Response: Done.

#### Composition of the Data

- *Edit sentence to read: “…Monitoring Committee consists of individuals…”*
- *Edit sentence to read: “…participant has been followed up for 84 days or discharged, whichever…”*

Response: Done.

#### Dissemination Plans

- *Low- and middle-income countries needs to be fully written out on first use, then the abbreviation “LMIC” can follow.*

Response: Done.

#### Discussion

- *Edit sentence to read: “…of its near absence from the global health agenda [25], and as such, very little…”*

Response: Done.

- *Edit sentence to read: “A Cochrane review [31] noted that previously published evidence is limited, due to a high or unclear risk of bias (selection, performance, detection, or attrition) detected, imprecision due to little participants, indirectness due to poor outcome measures, and inapplicable interventions.”*

Response: Done.

- *Edit sentence to read: “Although honey has been known for centuries to promoted wound healing, there are only a few controlled clinical trials that assess its efficacy.”*

Response: Done.

- *This would be a good place to include a brief discussion of relevant findings with specific outcomes or statistics, as I mentioned in the background section.*

### Anonymous [4]

#### General Comments

*This paper is a protocol description of an important study, especially for contexts in which advanced wound care products are often not available. It is a well-written protocol with clear steps to take. Below are some of my feedback; I also included some small textual feedback points in the text. You may not be able to address all the points I raised, as it seems that the trial has already started, but in that case, it would be interesting to describe why or why not in the manuscript’s text.*

#### Specific Comments

##### Major Comments

*1. Please describe why the 84-day cutoff period was chosen.*

Response: The choice of 84 days is based on a recent study by Rai et al [5], which suggests that 80% of leprosy ulcers were healed within 84 days following standard practice. This has been mentioned under the heading “Sample size.”

*2. The flow chart is a bit small and thus hard to read.*

Response: We have attached a full page of the flow chart as a supplementary material to make it easier for readers to understand.

*3. Usually, overlapping inclusion and exclusion criteria are not mentioned.*

Response: This is noted.

*4. It is not clear why hepatitis B or C were added in the exclusion criteria list.*

Response: We have considered that hepatitis B and C are not important confounders to the outcome of this study. We have now expunged the statement from the revised protocol.

*5. It is not clear for me if patients are clinically admitted or not, and if so, why? For how long? Is this routine care? And what are the discharge criteria?*

Response: All the study participants are hospitalized for up to 84 days or discharged when healing occurs before the 84-day period. Those whose ulcers are not healed after the 84-day period will continue with treatment but outside this study depending on the clinicians’ advice.

*6. If diabetes is excluded, it may be good to also exclude other known reasons for peripheral neuropathy (eg, vitamin B deficiencies).*

Response: This is an important suggestion. However, the study is already on course, and we have not considered vitamin B deficiencies from the inception. This is noted for future studies.

*7. Are signs of infection also monitored, assessed, or outcome measures?*

Response: Yes, the ulcers are beings observed for any sign of infection during the dressing changes. We report any serious adverse event throughout the study.

*8. Please explain more on the swabs: what kind of swab is it and what is tested, if this is not part of the research project? In general, it is better to take a routine swab to test for infection (bacterial growth) prior to inclusion instead of prior to randomization, as infection is an exclusion criteria. Also, address this under the heading about “biological specimens.”*

Response: Thank you for pointing this out. We have now addressed it to clearly show that wound swabs would be taken from the participants to screen for bacterial infections before recruitment. We have also reflected this in the section under “biological specimens.”

*9. Explain why the video recording is taking place. It may be interesting to also do it with the last 5 patients if it is performed for monitoring reasons.*

Response: The video recording is only for the purpose of quality control. The aim is for the independent assessors to verify that the protocol for the wound dressing is carefully followed. We also envisaged that this was only necessary at the start of the study.

*10. Why are assessors from Nepal used and not contextual assessors from Nigeria itself (also, is it because of skin color differences of participants in both countries)?*

Response: This is part of a multicountry study with India and Nepal. The plan was to send specimens across the countries to be examined by other researchers who understands the study but are completely blinded from the dressing allocation. For each ulcer, the measurement is carried out by 2 separate assessors, in which the measurements are later collated and harmonized by the data monitoring committee. We believe that, by doing so, the risk of bias will be greatly reduced.

*11. Please explain why early analysis is taking place after the inclusion of the first three-eighths of participants.*

Response: The purpose is to ensure that quality data are recorded throughout the study.

*12. I missed the argument in the discussion that mentioned that honey is often relatively cheap and better available than many advanced wound care products.*

Response: We have now mentioned that honey is cheaper and a readily available alternative to many wound care products.

*13. Include some information about how long data will be stored (number of years), where it will be stored in a secure way, and if it will be shared (pseudonymized) if requested (eg, for reproducibility).*

Response: The information is included under “Data management” and “Confidentiality.”

##### Minor Comments

*14. Write numbers up to 9 in text.*

Response: Noted.

*15. Check abbreviations.*

Response: All abbreviations checked.

*16. Update reference list, include authors, URLs, and “assessed on [date]” in references to websites and online documents.*

Response: Noted.

*17. Explain the camera used for photography.*

Response: The camera used is the Samsung Galaxy Tab S7 (13Mp) as mentioned in the protocol.

*18. Please add 2 more references in the discussion.*

Response: Noted.

*19. Explain more about the pedometer usage.*

Response: The pedometers are worn on the nonaffected foot of the participant to monitor their daily step counts. This might show if the level of activity or weight bearing has any impact on the healing rate of the ulcers.

## Abbreviations

REDCap: Research Electronic Data Capture
